# Evaluation of novel cathepsin-X inhibitors in vitro and in vivo and their ability to improve cathepsin-B-directed antitumor therapy

**DOI:** 10.1007/s00018-021-04117-w

**Published:** 2022-01-06

**Authors:** Ana Mitrović, Janja Završnik, Georgy Mikhaylov, Damijan Knez, Urša Pečar Fonović, Petra Matjan Štefin, Miha Butinar, Stanislav Gobec, Boris Turk, Janko Kos

**Affiliations:** 1 Department of Biotechnology, Jožef Stefan Institute, Jamova 39, 1000 Ljubljana,, Slovenia; 2 Department of Biochemistry and Molecular Biology, Jožef Stefan Institute, Ljubljana, Slovenia; 3 Faculty of Pharmacy, University of Ljubljana, Ljubljana, Slovenia; 4 Faculty of Chemistry and Chemical Technology, University of Ljubljana, Ljubljana, Slovenia

**Keywords:** Cathepsin X, Cathepsin B, Inhibitors, Cancer, Antitumor therapy, Invasion

## Abstract

New therapeutic targets that could improve current antitumor therapy and overcome cancer resistance are urgently needed. Promising candidates are lysosomal cysteine cathepsins, proteolytical enzymes involved in various critical steps during cancer progression. Among them, cathepsin X, which acts solely as a carboxypeptidase, has received much attention. Our results indicate that the triazole-based selective reversible inhibitor of cathepsin X named Z9 (1-(2,3-dihydrobenzo[*b*][1,4]dioxin-6-yl)-2-((4-isopropyl-4*H*-1,2,4-triazol-3-yl)thio)ethan-1-one) significantly reduces tumor progression, both in vitro in cell-based functional assays and in vivo in two independent tumor mouse models: the FVB/PyMT transgenic and MMTV-PyMT orthotopic breast cancer mouse models. One of the mechanisms by which cathepsin X contributes to cancer progression is the compensation of cathepsin-B activity loss. Our results confirm that cathepsin-B inhibition is compensated by an increase in cathepsin X activity and protein levels. Furthermore, the simultaneous inhibition of both cathepsins B and X with potent, selective, reversible inhibitors exerted a synergistic effect in impairing processes of tumor progression in in vitro cell-based assays of tumor cell migration and spheroid growth. Taken together, our data demonstrate that Z9 impairs tumor progression both in vitro and in vivo and can be used in combination with other peptidase inhibitors as an innovative approach to overcome resistance to antipeptidase therapy.

**Supplementary Information:**

The online version contains supplementary material available at 10.1007/s00018-021-04117-w.

## Introduction

Despite tremendous progress in cancer treatment during the last decades, cancer remains one of the leading causes of death in the developed world [1]. Therefore, identifying new therapeutic targets and developing new approaches are crucial to further improve antitumor therapy and combat cancer [2]. An important role in cancer progression is attributed to proteolytic enzymes [3, 4], among which a group of lysosomal cysteine peptidases deserves special attention. In cancer, cysteine cathepsins are overexpressed, translocated from lysosomes and endosomes to the cell membrane or secreted from cells, and involved in various stages of cancer development and progression, including extracellular matrix (ECM) degradation, cell invasion, cell migration, metastasis, and angiogenesis [5]. Cathepsin X (CatX) differs from other cysteine cathepsins due to its very short proregion and a three-residue long insertion motif that forms a characteristic “mini loop” defining its exclusive carboxypeptidase activity [6, 7]. Additionally, CatX is incapable of autoactivation and must be activated by endopeptidases, such as cathepsin L or E, within a proteolytic cascade [7, 8].

CatX is predominantly expressed by immune and neuronal cells [6]. In immune cells, it regulates cell migration, proliferation, maturation, adhesion, phagocytosis, and signal transduction through interaction with integrin receptors [6, 9]. In brain tissue, CatX eliminates the neurotrophic activity of γ-enolase by cleaving two amino acid residues at its C-terminus [10], and is proposed to have an effect on neuron survival and neurite outgrowth [11, 12]. Several other molecular targets have been identified as substrates of CatX, including chemokine CXCL-12, bradykinin, kallidin, huntingtin, and profilin-1 [6].

Increased CatX protein levels and activity have been found in various cancers [6, 13–17], correlating with shorter patient survival or poor disease outcome [18, 19]. The promotive role of CatX in cancer is consistent with the location of its gene, in chromosomal region 20q13, which is frequently amplified in various cancers [20]. Several mechanisms have further link CatX to tumor progression, including bypassing senescence, modulating adhesion and migration of tumor cells through cleavage of integrin receptors and profilin-1, and inducing epithelial-mesenchymal transition [6].

Moreover, it has been proposed that CatX compensates for tumor-associated decreases in cathepsin-B (CatB) activity, as loss of CatB was followed by increased CatX expression and activity [21, 22]. This compensatory effect was first observed in a cathepsin-B/-X-deficient transgenic polyoma middle T oncogene (PyMT)-induced breast cancer mouse model. Dual deficiency of both cathepsins B and X resulted in synergistic antitumor effects leading to significantly reduced tumor growth and metastasis formation. Deficiency of CatB alone resulted in delayed detection of initial tumors and reduced tumor burden, however, only up to a certain stage. Deficiency was followed by increased CatX expression, which compensated for the loss of CatB [21, 22]. Furthermore, a separate study demonstrated that double knockout of cathepsins B and X is necessary to reduce laser-induced choroidal neovascularization, supporting the existence of a compensatory mechanism between related cathepsins also during angiogenesis [23]. Moreover, our group confirmed the redundant function between the two cathepsins and the compensatory role in epithelial–mesenchymal transition following the induction of the reverse process, i.e., mesenchymal–epithelial transition, after concurrent silencing of both cathepsins B and X in the lung carcinoma cell line A549 [24]. In addition to cancer, a compensatory role between cathepsins B and X has also been suggested in inflammation [25].

Therefore, CatX may be considered an additional therapeutic target that can interfere with the excessive proteolytic activity observed in cancer [6]. However, the number of known specific CatX inhibitors is rather small. Endogenous inhibitors of CatX are not known [26]. Besides the neutralizing antibody 2F12 [16], only the irreversible epoxysuccinyl inhibitor AMS-36 [27] was available until recently. In our previous study, we identified triazole-based compounds as potent, reversible, selective inhibitors of CatX [28]. Among them, the compound **Z9** (1-(2,3-dihydrobenzo[*b*][1,4]dioxin-6-yl)-2-((4-isopropyl-4*H*-1,2,4-triazol-3-yl)thio)ethan-1-one, also designated as compound **22** in our previous publication [28]) had the lowest inhibition constant (2.45 ± 0.05 μM) and showed good selectivity for CatX compared to the cysteine cathepsins B, L, H, and S. Further investigation of chemical variations of **Z9** revealed that the central ketomethylenethio linker connecting the benzodioxine and triazole moieties was crucial for CatX inhibition, whereas changes in triazole heterocycle did not alter the inhibitory potency. Finally, replacement of benzodioxine with the substituted benzenes reduced the inhibition of CatX [29]. Preliminary studies showed that non-cytotoxic concentrations of **Z9** may impair tumor cell migration and promote neurite outgrowth [28].

In the present study, we aimed to further evaluate the antitumor properties of **Z9** by assessing its effect on tumor growth, migration, and invasion in vitro and in vivo. In addition, we investigated the ability of CatX inhibition to interfere with the compensation between cathepsins B and X, which might prevent tumor resistance during antipeptidase therapy.

## Materials and methods

### Inhibitors

Compound **Z9** was synthesized as reported previously [29], nitroxoline was obtained from Sigma-Aldrich (St. Luis, MO, USA), and CA-074 was from Peptide Institute Inc. (Osaka, Japan).

### Cell lines

MCF-10A neoT, a human mammary epithelial cell line transfected with the c-Ha-ras oncogene, was provided by Bonnie F. Sloane (Wayne State University, Detroit, MI). Primary tumor cells isolated from mammary carcinomas that spontaneously develop in mouse mammary virus-polyoma middle T antigen (MMTV-PyMT) transgenic mice were isolated and cultured as described [21]. MCF-10A neoT cells were cultured in Dulbecco’s modified Eagle’s medium (DMEM)/Nutrient Mixture F-12 (1:1) medium with GlutaMAX™ (Gibco, Carlsbad, CA, USA) supplemented with 5% fetal bovine serum (FBS, Gibco), 1 µg/mL insulin (Sigma-Aldrich), 0.5 µg/mL hydrocortisone (Sigma-Aldrich), 20 ng/mL epithelial growth factor (Milipore, Burlington, MA, USA), and 1% penicillin–streptomycin (Gibco), corresponding to 100 U/mL penicillin and 100 µg/mL streptomycin. MMTV-PyMT cells were cultured in DMEM with GlutaMAX™ (Gibco) supplemented with 10% FBS, and 1% penicillin–streptomycin (all Gibco). Cells were maintained at 37 °C in a humidified atmosphere with 5% CO_2_ and cultured until they reached 80% confluency. Prior to use, cells were detached from culture flasks with TripLE™ Select Enzyme (Gibco).

### DQ-collagen IV degradation assay

DQ-collagen IV was used to observe the effect of CatB and CatX inhibition on EMC degradation. Intracellular degradation of DQ-collagen type IV was monitored by flow cytometry and extracellular degradation was monitored with spectrofluorimetry. The detailed protocols are provided in Supplementary Methods.

### Scratch assay

The scratch assay was used to monitor tumor cell migration. Briefly, 2.5 × 10^4^ MCF-10A neoT cells/well and 8 × 10^5^ MMTV-PyMT cells/well were seeded into a 6-well plate and allowed to adhere and form a confluent layer overnight at 37 °C. The medium was then replaced with fresh SFM or medium, respectively, containing **Z9**, nitroxoline, both nitroxoline and **Z9** (all 5 µM), or DMSO (0.1%). After 2 h of incubation at 37 °C, the cell monolayer was scraped using a 200 µL pipette tip. Images were acquired at 0 and 16 h (for MCF-10A neoT cells) or 24 h (for MMTV-PyMT cells) on an Olympus CKX 41 light microscope with an Olympus E-450 camera (Olympus, Tokyo, Japan) at 10× magnification. The scratch area was determined using ImageJ software with the wound-healing size tool (https://github.com/AlejandraArnedo/Wound-healing-size-tool/wiki) [30]. Cell migration was expressed as a percentage of scratch area and calculated using the following equation: Area (%) = 100 × (*A*_*t*_/*A*_0_), where *A*_*t*_ and *A*_0_ denote gap area at time *t* (16 or 24 h) and the initial time point, respectively. For each individual treatment condition, at least two images with different fields of view were acquired and five independent replications of experiment were performed.

### Real-time migration, invasion, and adhesion assays

Tumor cell migration, invasion, and adhesion were monitored in real-time with an xCELLigence Real-Time Cell Analyzer (RTCA; Agilent, Santa Clara, CA, USA). The detailed protocol is provided in Supplementary Methods.

### Three-dimensional invasion assay

The effect of cathepsin-B and -X inhibition on tumor cell invasion in a three-dimensional (3D) model was evaluated using multicellular tumor spheroids implemented in Matrigel, representing a model of the ECM. The detailed protocol is provided in Supplementary Methods.

### Mouse tumor models

Animal experiments were performed in accordance with EU directives and approval from the Veterinary Administration of the Ministry of Agriculture, Forestry and Food of the Republic of Slovenia (approval number: #U34401-28/2017/5). Mice were housed in a specific pathogen-free animal colony at controlled temperature and humidity with a 12 h light–dark cycle. Food and water were provided ad libitum.

### MMTV-PyMT mouse breast cancer model

To address the effects of the different compounds on mammary gland tumor formation and lung metastases, FVB/N transgenic mice expressing the PyMT oncogene under the control of the MMTV LTR promoter (FVB/N-TgN (MMTV-PyMT)634-Mul) were used [31]. At 11 weeks of age, the transgenic mice were randomly divided into four groups and administered: (1) only vehicle (5% DMSO in peanut oil; *n* = 10), (2) **Z9** (67.18 mg/kg, equal in molar concentration to the dose of nitroxoline; *n* = 10), (3) nitroxoline (40 mg/kg; *n* = 10), or (4) both nitroxoline (40 mg/kg) and **Z9** (67.18 mg/kg) (*n* = 10). The treatment was injected intraperitoneally every other day, and the mice were sacrificed at 14 weeks of age. Mammary tumor tissue was collected and weighed, and the lungs were dissected and processed for histomorphometric analysis.

### Orthotopic mouse model

The orthotopic mouse model was established in FVB/N mice by inoculating 5 × 10^5^ primary MMTV-PyMT tumor cells in SFM into the left inguinal mammary gland of FVB mice [32]. Tumor-bearing FVB mice were randomly divided into four groups and administered: (1) only vehicle (5% DMSO in peanut oil; *n* = 6), (2) **Z9** (33.59 mg/kg; *n* = 6), (3) nitroxoline (20 mg/kg; *n* = 6), or (4) both nitroxoline (20 mg/kg) and **Z9** (33.59 mg/kg; *n* = 6). Treatment began when the tumor volume reached 125 mm^3^ and was injected intraperitoneally every other day. Throughout the course of treatment, the tumor dimensions were measured every other day, and the tumor volume was calculated according to the equation: *V* = (π × (tumor width)^2^ × (tumor length))/6.

### Statistical analysis

The GraphPad Prism 6.0 software package (GraphPad Software, San Diego, USA) was used for data analysis. Data are presented as means ± SEM unless stated otherwise. Statistically significant differences between data groups were assessed using the nonparametric, two-tailed Student’s *t* test, unless stated otherwise. Differences were considered significant at *P* ≤ 0.05.

## Results

### CatX expression and activity are increased after CatB inhibition

**Fig. 1:**
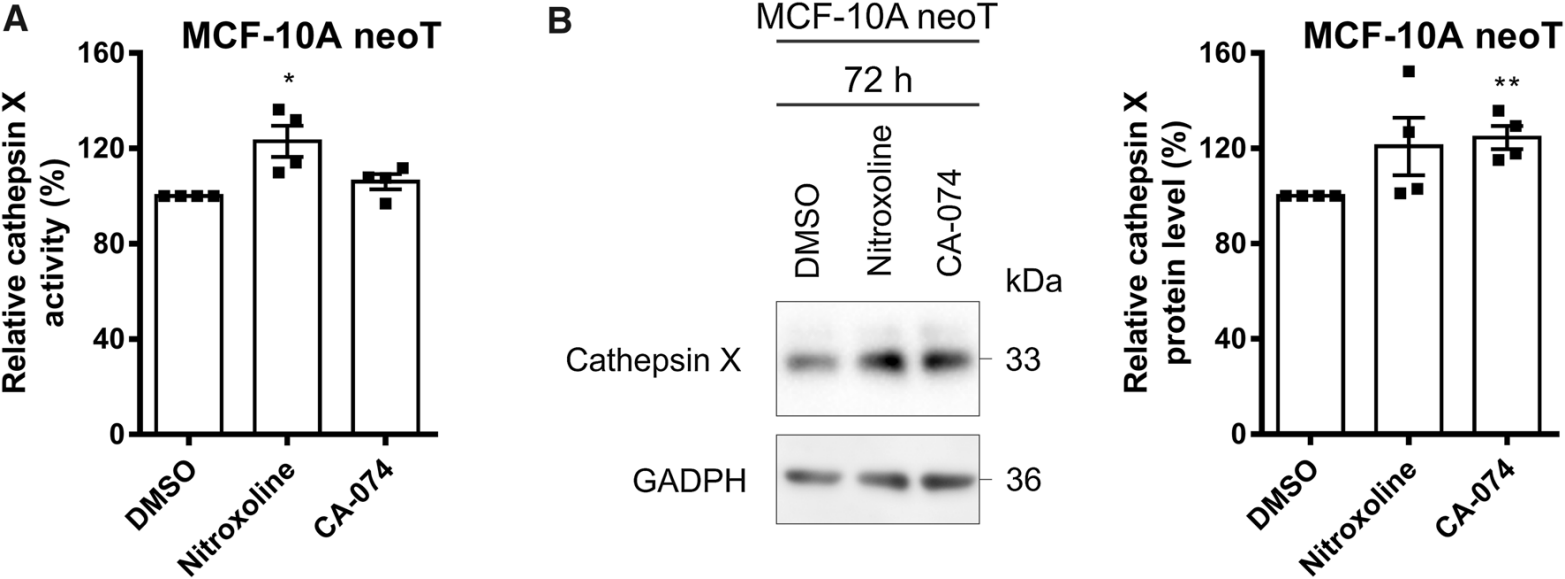
CatX protein levels and activity are increased after CatB inhibition. **A** After 48 h of incubation of MCF-10A neoT cells (1.5 × 10^5^) with nitroxoline (5 µM) or CA-074 (5 µM), CatX activity is increased compared to that of control cells treated with DMSO (0.05%). **B** CatX protein levels in MCF-10A neoT cells after 72 h of incubation with nitroxoline (5 µM), CA-074 (5 µM), or DMSO (0.05%). Protein levels were determined by western blot analysis. The histogram shows the relative CatX protein levels. Data are presented as mean ± SEM (*n* = 4). *P < 0.05, **P < 0.01

To assess the compensatory effect between cathepsins B and X, we first tested how CatB inhibition affects CatX expression. MCF-10A neoT cells expressing both cathepsins at high levels were treated with the CatB inhibitors nitroxoline (5 µM) or CA-074 (5 µM) or DMSO (0.05%). CatX activity was increased by 23.1 ± 6.5% and 6.1 ± 3.2% after 48 h of treatment with nitroxoline and CA-074, respectively (Fig. 1A), while its protein levels were increased by 20.8 ± 12.0% and 24.5 ± 4.9% after 72 h of treatment with nitroxoline and CA-074, respectively (Fig. 1B).

### Z9 alone or in combination with nitroxoline does not affect the viability of tumor cells

First, to rule out the possibility that any effects of the compounds in the functional assays could be due to compound-induced toxicity, we examined the effects of **Z9** (Supp. Figure 1A) alone and in combination with nitroxoline on cell viability using the MTS cell viability assay. The viability of MCF-10A neoT (Supp. Figure 1B) or MMTV-PyMT cells (Supp. Figure 1C) was not essentially reduced after treatment with **Z9** at concentrations up to 10 µM for 24, 48, or 72 h. Additionally, cell viability was examined after co-treatment with nitroxoline and **Z9** (Supp. Figure 1A) at concentrations up to 5 µM each for 24, 48, or 72 h. Cell viability was not affected in either cell line after 24 h of treatment; however, cell viability decreased only slightly after 72 h of co-treatment with both compounds (at 5 µM). Based on these results and considering the incubation times where the effects of compounds were monitored, both compounds were used at concentrations of 5 µM in further experiments.

### Inhibition of CatB, but not CatX, impairs degradation of ECM by tumor cells

**Fig. 2:**
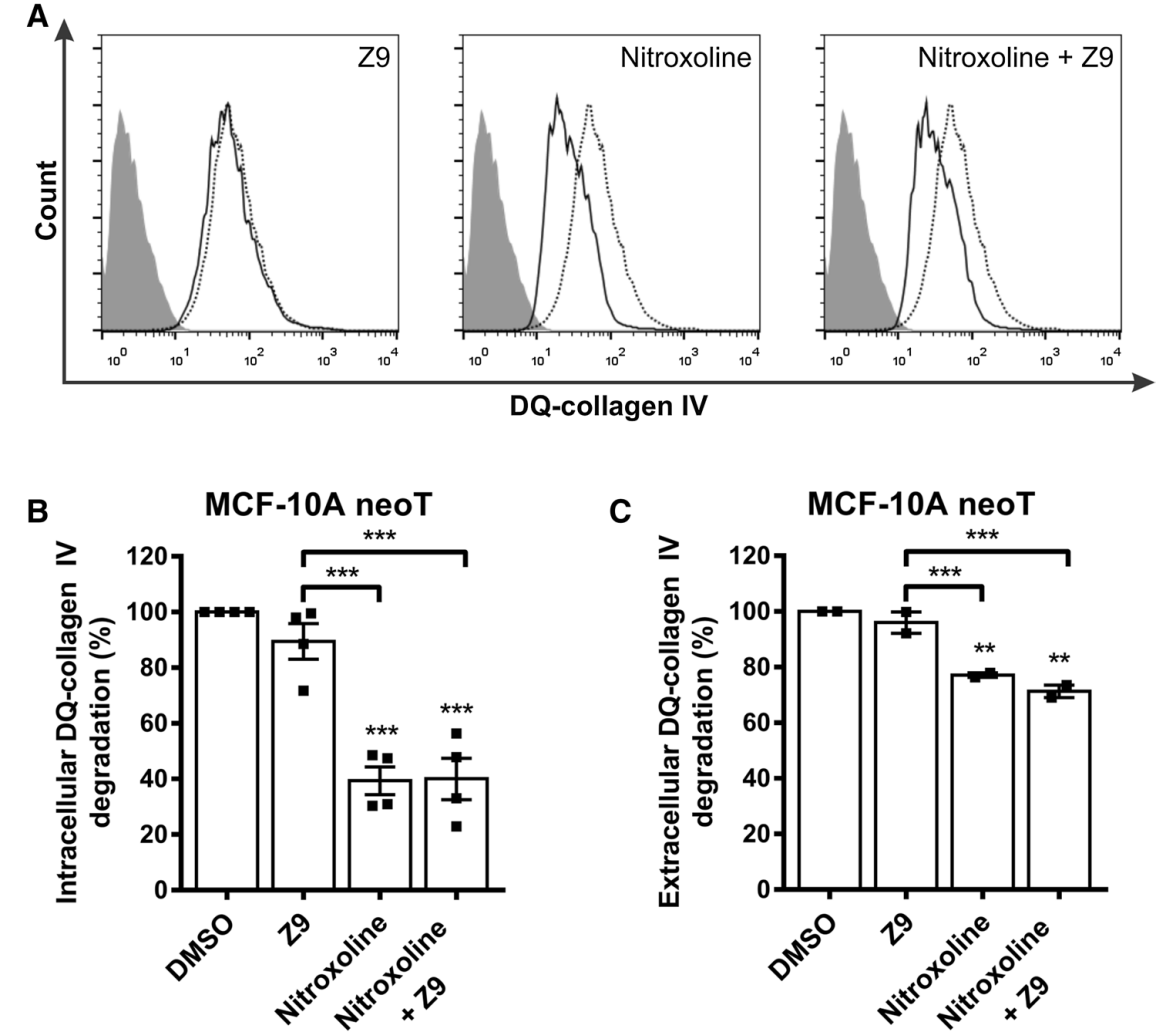
CatX inhibition does not affect intracellular or extracellular degradation of ECM. **A** Intracellular DQ-collagen IV degradation by MCF-10A neoT cells (6 × 10^4^) after treatment with DMSO (0.5%, dotted black line) or the compounds (solid black line) **Z9** (50 μM), nitroxoline (50 μM), or both nitroxoline and **Z9** (50 μM each), as monitored by flow cytometry. Gray histograms denote unlabeled cells. **B** Reduced intracellular DQ-collagen IV degradation in the presence of CatB and CatX inhibitors compared to DMSO, calculated from data obtained by flow cytometry. Data are presented as mean ± SEM of four independent experiments, each performed in duplicate. **C** Reduced extracellular DQ-collagen IV degradation by MCF-10A neoT cells (3 × 10^4^) in the presence of CatB and CatX inhibitors **Z9** (5 μM), nitroxoline (5 μM), or both nitroxoline and **Z9** (5 μM each) compared to DMSO (0.1%) was analyzed by monitoring the fluorescence intensity of the extracellular product of DQ-collagen IV degradation. Data are presented as means ± SEM (*n* = 2). *P < 0.05, **P < 0.01, ***P < 0.001

One of the most crucial processes during tumor progression that promotes tumor cell invasion and metastasis is the degradation of ECM. Cells can degrade ECM either extracellularly by secreted and membrane-associated proteases or intracellularly in lysosomes after endocytosis of partially degraded ECM components [33–35]. The effects of cathepsins B and X on the degradation of ECM were investigated by studying the degradation of DQ-collagen IV by MCF-10A neoT cells, which were previously shown to degrade DQ-collagen IV both intracellularly and extracellularly [36]. Collagen IV is a major component of the ECM and can be fluorescently labeled, thus fluorescing bright green upon proteolytic cleavage. Intracellular and extracellular degradation of DQ-collagen IV was quantified by flow cytometry and spectrofluorimetry, respectively (Fig. 2). The cathepsin-B-specific inhibitor nitroxoline and the combination of the cathepsin-B- and -X-specific inhibitors nitroxoline and **Z9**, respectively, significantly reduced the degradation of DQ-collagen IV by 60.7 ± 5.0% and 60.0 ± 7.5%, respectively, whereas the CatX inhibitor **Z9** alone reduced the degradation of DQ-collagen IV by only 10.5 ± 6.4% (Fig. 2B). A similar trend was also observed for extracellular degradation of DQ-collagen IV; nitroxoline and both compounds together reduced degradation by 22.9 ± 0.8% and 28.7 ± 2.2%, respectively, while **Z9** reduced degradation by only 4.0 ± 3.8% (Fig. 2C).

### Simultaneous inhibition of cathepsins B and X synergistically decreases tumor cell migration

**Fig. 3:**
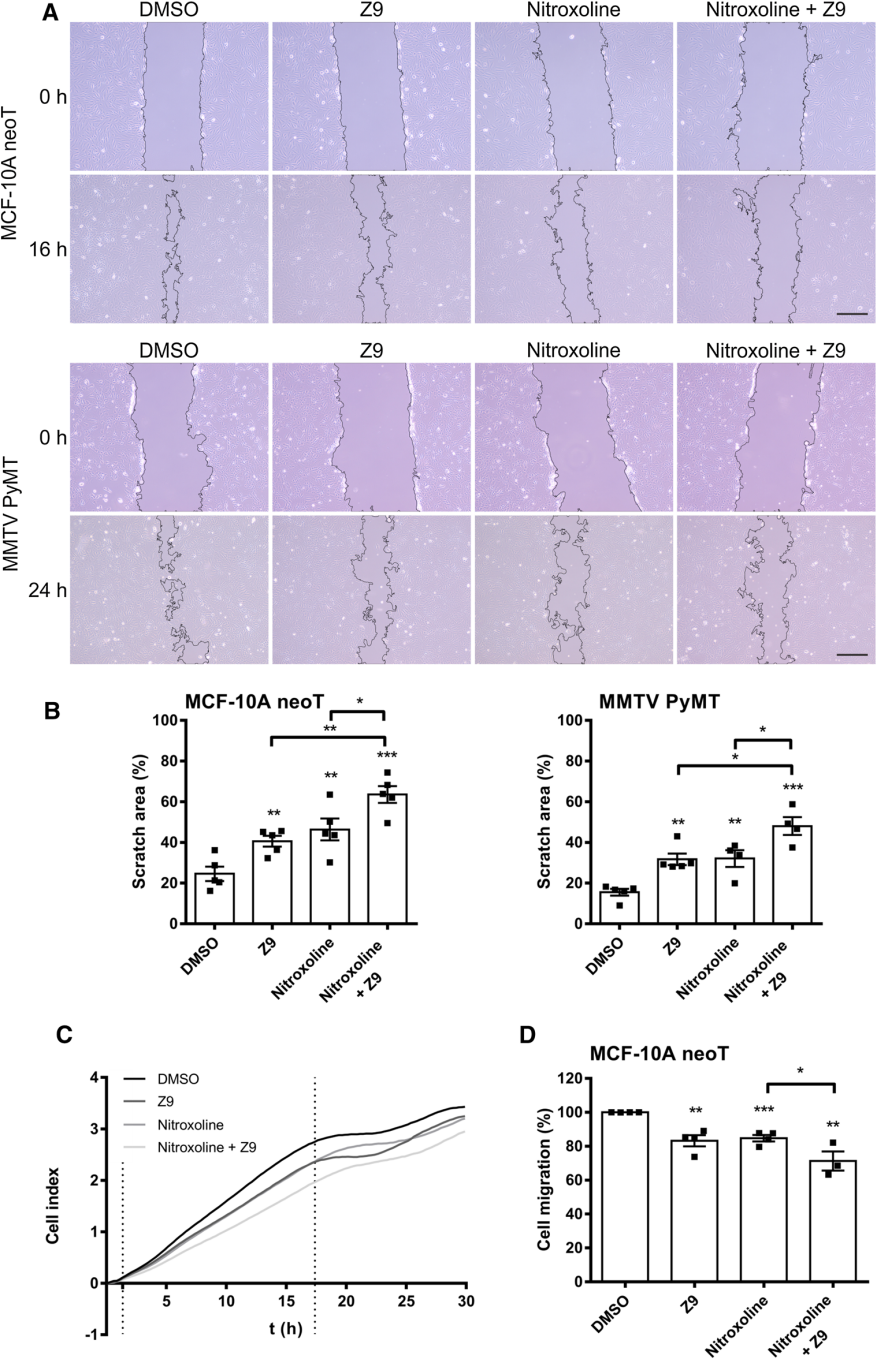
The simultaneous inhibition of cathepsins B and X further impairs tumor cell migration. **A** In the in vitro scratch assay, confluent monolayers of MCF-10A neoT (2.5 × 10^5^) and MMTV-PyMT (8 × 10^5^) cells treated with either **Z9** (5 μM), nitroxoline (5 μM), both nitroxoline and **Z9** (5 μM each), or DMSO (0.1%) were scratched, and the migration of tumor cells was monitored as scratch closure. Images of the scratches were taken on a light microscope at 10× magnification after 0 and 16 h (for MCF-10A neoT cells) or 24 h (for MMTV-PyMT cells). Scale bar: 250 μm. **B** Scratch areas were quantified using ImageJ software with the Wound Healing Size tool, and cell migration was expressed as percentage of scratch area (%). Data are presented as mean ± SEM. Points represent the average of at least two images with different fields of view acquired per individual treatment condition. **C** Tumor cell migration in real time, monitored by the xCELLigence system. MCF-10A neoT cells (2 × 10^4^) were seeded in the upper chambers, and migration was monitored continuously by measuring impedance data [reported as cell index (CI)]. The plot shows representative curves of cell migration in the presence of **Z9** (5 μM), nitroxoline (2.5 μM), both nitroxoline (2.5 μM) and **Z9** (5 μM), or DMSO (0.1%). **D** Percentages of migrated cells were calculated from the slopes of the CI as a function of time in the time interval indicated by the vertical lines in (**C**). Data are presented as mean ± SEM (*n* = 3, each in triplicate). *P < 0.05, **P < 0.01, ***P < 0.001

The ability of CatX inhibition and concomitant CatB and CatX inhibition to reduce tumor cell migration was examined with the scratch assay (for MCF-10A neoT and MMTV-PyMT cells) and the xCELLigence system (for MCF-10A neoT cells). The scratch assay is a simple and widely used in vitro technique that assesses cell migration in response to various treatments by monitoring gap closure. The larger scratch areas observed in the presence of the inhibitors after 16 and 24 h for MCF-10A neoT and MMTV-PyMT cells, respectively, indicate that **Z9**, nitroxoline, and their co-treatment significantly decrease cell migration (Fig. 3A). For MCF-10A neoT cells, the scratch areas after 16 h were 40.6 ± 2.7% (5 μM **Z9**), 46.4 ± 5.4% (5 μM nitroxoline), 63.6 ± 4.1% (both 5 μM nitroxoline and **Z9**), and 24.6 ± 3.5% (DMSO) (Fig. 3B). For MMTV-PyMT cells, the scratch areas after 24 h were 31.7 ± 2.8% (5 μM **Z9**), 32.1 ± 4.1% (5 μM nitroxoline), 40.1 ± 4.3% (both 5 μM nitroxoline and **Z9**), and only 15.5 ± 1.7% (DMSO) (Fig. 3B). In both cell lines, the scratch areas were significantly larger after co-treatment compared to the scratch areas in the presence of a single inhibitor.

Next, the effect on tumor cell migration was examined in real time using the xCELLigence system (Fig. 3C). Similar to the scratch assay, both inhibitors significantly decreased the migration of MCF-10A neoT cells. Tumor cell migration was reduced by 16.7 ± 6.6% (5 μM **Z9**), 15.2 ± 3.8% (2.5 μM nitroxoline), and 22.9 ± 9.3% (2.5 μM nitroxoline and 5 μM **Z9)** (Fig. 3D). Moreover, co-treatment significantly enhanced the inhibition of cell migration compared to nitroxoline alone. However, regardless of the stronger inhibition after co-treatment, co-treatment did not significantly decrease migration compared to 5 μM **Z9** alone.

### Simultaneous inhibition of cathepsins B and X enhances the inhibition of tumor cell invasion in vitro

**Fig. 4:**
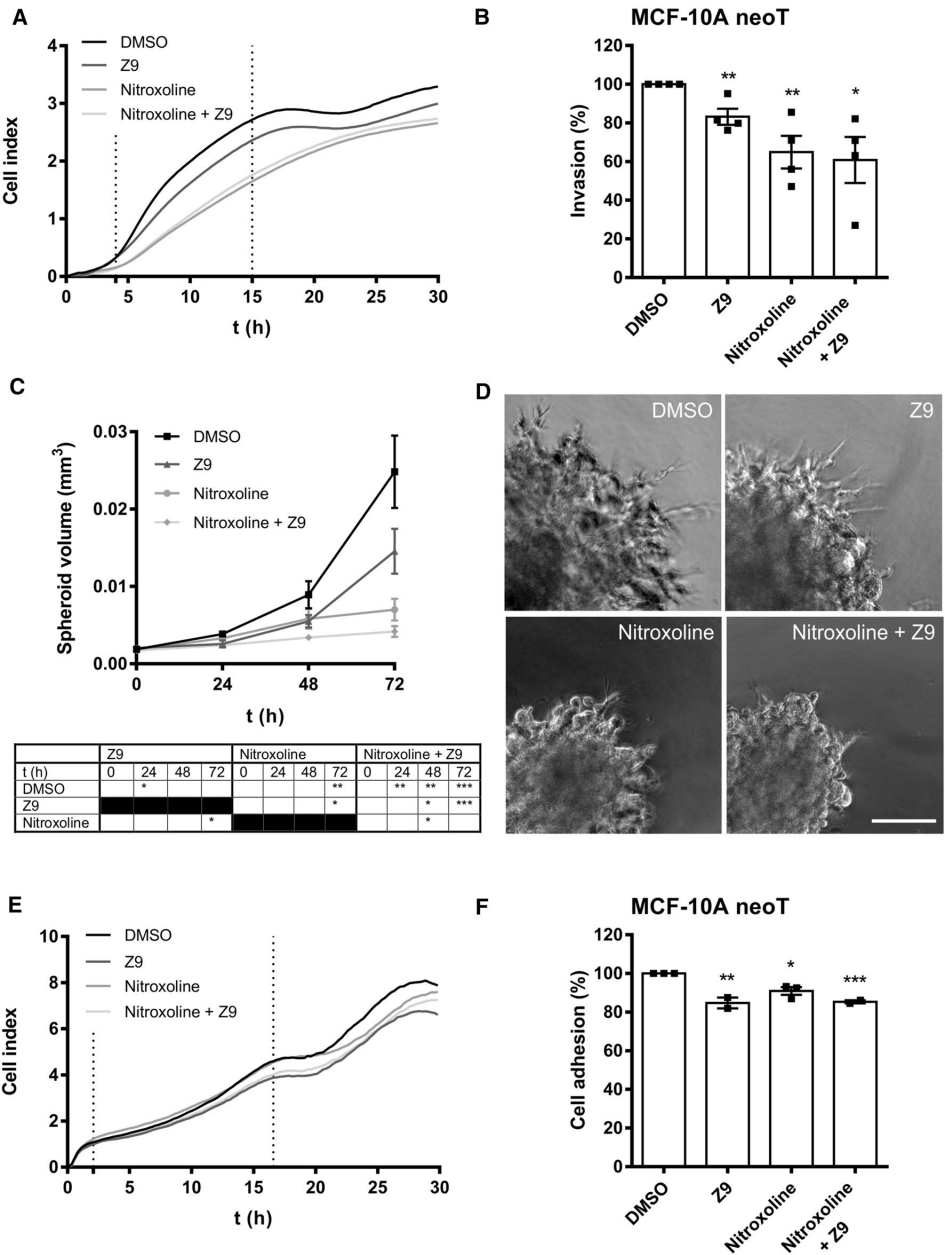
Tumor cell invasion and adhesion is impaired by the inhibition of cathepsins B and X. **A** MCF-10A neoT cells (3 × 10^4^) were seeded onto Matrigel-coated wells, and the invasion from the top chamber to the bottom chamber driven by serum gradient in the presence of DMSO (0.1%), **Z9** (5 μM), nitroxoline (5 μM), and both nitroxoline and **Z9** (5 μM each) together was monitored with the xCELLigence system. The curves represent the invasion of cells observed as changes in cell index versus time. **B** The slopes in the time interval indicated by the vertical lines in (**A**) were used to calculate the percentage of invasion, represented as mean ± SEM (*n* = 4, each in triplicate). **C** The growth of MMTV-PyMT spheroids implanted in Matrigel containing **Z9** (5 μM), nitroxoline (5 μM), both nitroxoline and **Z9** (5 μM each), or DMSO (0.1%) was measured by measuring the spheroid dimensions under an optical microscope with an ocular micrometer for up to 3 days. Data are presented as mean ± SEM (*n* = 3). **D** Representative images of MMTV-PyMT spheroids 3 days after implantation. Scale bar: 100 μm. **E** The reduction of tumor cell adhesion in the presence of CatB and CatX inhibitors. Adhesion of tumor cells in real time was observed with the xCELLigence system. MCF-10A neoT cells (5 × 10^3^) were seeded into the fibronectin-coated wells, and the adhesion of the cells to the bottom of the wells was monitored continuously by measuring cell index. The plot shows representative curves of the adhesion of cells treated with **Z9** (5 μM), nitroxoline (2.5 μM), both nitroxoline (2.5 μM) and **Z9** (5 μM) together, or DMSO (0.1%). **F** The slopes of the cell index as a function of time in the time interval indicated by the vertical lines in (**E**) were used to calculate the relative percentage of adhered cells. Data are presented as mean ± SEM. The experiment was performed in triplicate and repeated at least two times. *P < 0.05, **P < 0.01, ***P < 0.001

The effects of CatB and CatX inhibition on tumor cell invasion were first assessed by continuously monitoring the invasion of MCF-10A neoT cells through a Matrigel layer, which is a model for the ECM, using the xCELLigence system (Fig. 4A). The invasion of MCF-10A neoT cells was significantly reduced by 16.8 ± 4.1% (5 μM **Z9**), 35.1 ± 8.4% (5 μM nitroxoline), and 39.2 ± 11.9% (5 μM nitroxoline and **Z9**) (Fig. 4B). Although tumor cell invasion was most inhibited after co-treatment, the synergistic effect of both compounds together versus the single compounds was not statistically significant in this experiment.

Next, to further evaluate the effects of the inhibitors on tumor cell invasion, a 3D in vitro tumor cell invasion model was used. The model is based on implantation of multicellular tumor spheroids into Matrigel (a model of ECM), thus mimicking early avascular stages of tumor growth. This model represents an upgrade over classical models in which cell invasion is monitored in monolayers. For this purpose, MCF-10A neoT and MMTV-PyMT spheroids were prepared according to the hanging drop method and implanted into Matrigel to monitor the effect of inhibitors on tumor cell invasion. Unfortunately, the MCF-10A neoT spheroids failed to invade the Matrigel, as no radially invading strands and invasive corona around the original spheroid were formed enabling monitoring of the effect of inhibitors on tumor cell invasion. Therefore, only MMTV-PyMT spheroids, which formed radially invading strands in a sunburst pattern and showed uniform growth through the course of the experiment, were used to monitor the effect of CatB and CatX inhibition on tumor cell invasion in the 3D model (Fig. 4C, D). After 72 h of incubation, nitroxoline (5 μM) and the combination of nitroxoline and **Z9** (5 μM each) significantly reduced spheroid growth by 71.7 ± 5.7% and 81.8 ± 3.0%, respectively, compared to control cells (Fig. 4C). Furthermore, after 72 h, **Z9** (5 μM) alone reduced spheroid growth by 41.4 ± 11.8%; however, the decrease in spheroid growth was not significant compared to control spheroids. Moreover, co-treatment significantly decreased spheroid growth compared to **Z9** alone at 48 and 72 h, and compared to nitroxoline at 48 h. The reduced invasion of tumor cells from the original spheroid and their reduced growth in the presence of the inhibitors compared to control cells was also observed on the representative images obtained 72 h after spheroid implantation (Fig. 4D).

### The effect of cathepsins B and X on tumor cell adhesion

Similarly, as for tumor cell migration and invasion, the xCELLigence system was used to continuously monitor tumor cell adhesion. Both **Z9** (5 μM) and nitroxoline (2.5 μM) as well as co-treatment significantly reduced the adhesion of MCF-10A neoT cells compared to control cells (Fig. 4E). Cell adhesion was reduced by 15.3 ± 2.8% (5 μM **Z9**), 9.1 ± 2.0% (2.5 μM nitroxoline), and 14.7 ± 0.6% (2.5 μM nitroxoline and 5 μM **Z9**) (Fig. 4F). However, co-treatment showed no significant reduction in tumor cell adhesion compared to individual treatment with the CatB or X inhibitors.

### CatX inhibition impairs tumor progression in vivo

**Fig. 5:**
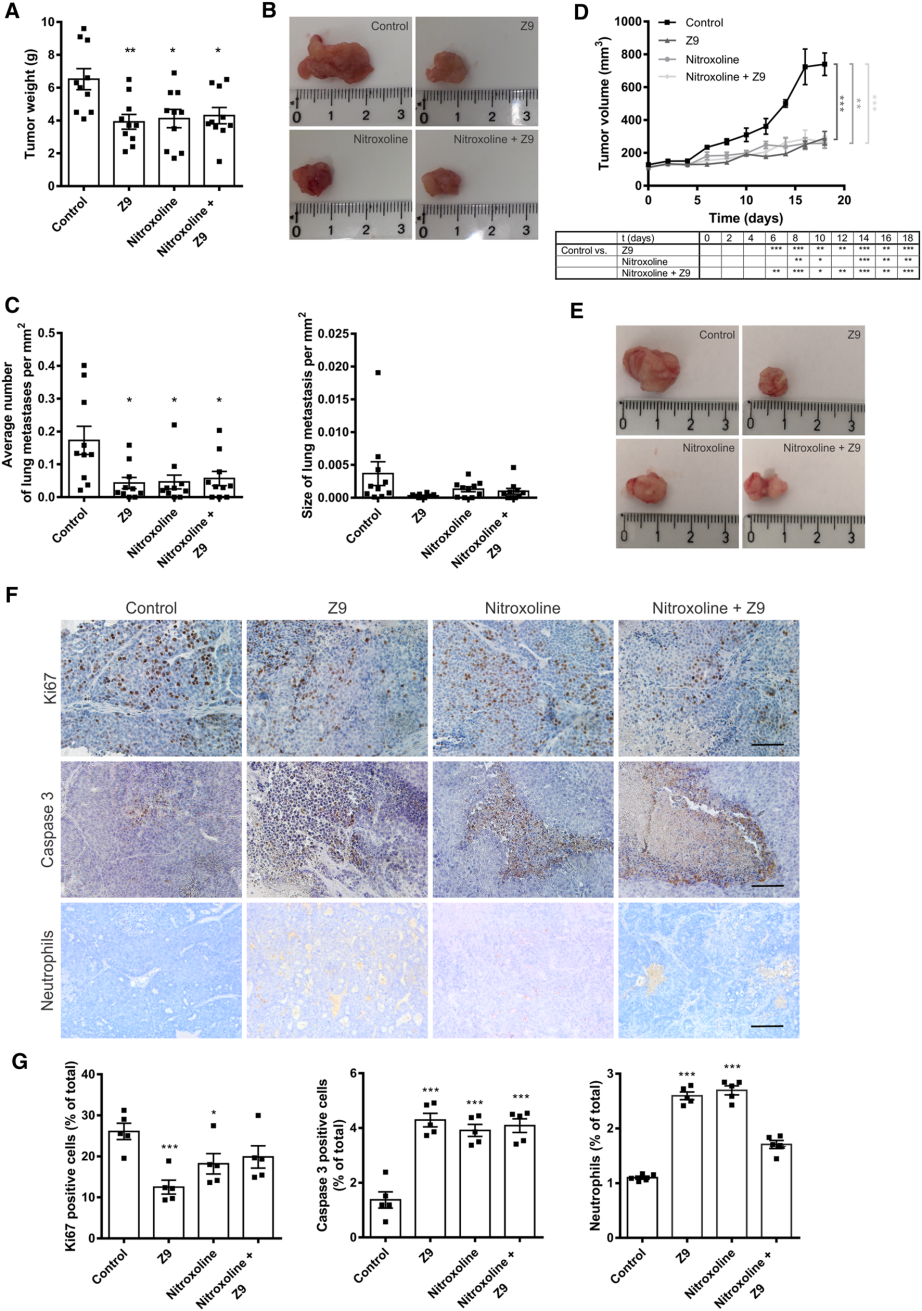
**Z9** impairs tumor progression in vivo. **A** Z9 impairs tumor growth and metastasis in the FVB/PyMT transgenic mouse model, where transgenic 11-week-old PyMT mice were treated every other day with **Z9** (67.18 mg/kg; *n* = 10), nitroxoline (40 mg/kg; *n* = 10), or simultaneously with nitroxoline (40 mg/kg) and **Z9** (67.18 mg/kg; *n* = 10). The control group (*n* = 10) was injected with vehicle (5% DMSO in peanut oil). At 14 weeks of age, the mice were sacrificed, and the mammary tumors were collected and weighed. **B** Representative images of individual tumors obtained from FVB/PyMT transgenic mice for each group. **C** To determine the effect of treatment on pulmonary metastasis from FVB/PyMT transgenic mouse, lungs were dissected and analyzed. Sections (5 µm thick) of paraffin-embedded lungs were stained with hematoxylin and eosin, and the number and size of pulmonary metastases were determined. Data are presented as mean ± SEM. **D** **Z9** reduced tumor growth in an orthotopic mouse breast cancer model that was established by introducing MMTV-PyMT cells (5 × 10^5^) into the left inguinal mammary gland of FVB mice. When the tumor volume reached 125 mm^3^, the mice were treated every other day with **Z9** (33.59 mg/kg; *n* = 6), nitroxoline (20 mg/kg; *n* = 6), or with both nitroxoline (20 mg/kg) and **Z9** (33.59 mg/kg; *n* = 6). The control group (*n* = 6) was injected with vehicle (5% DMSO in peanut oil). The tumor volume was continuously monitored every other day throughout the course of the experiment. The table shows the significant differences between the mice treated with inhibitors vs. control. **E** Representative images of tumors from orthotopic mouse breast cancer model for each group. **F** The effect of CatX and B inhibition on tumor cell proliferation and death and neutrophil infiltration in an orthotopic mouse tumor model. Cell proliferation of tumor cells as determined by immunodetection of Ki67, cell death as determined by immunodetection of caspase 3, and infiltration of neutrophils into tumor tissue. Representative images were taken under the IX81 bright-field microscope (Olympus) at 40× magnification. Scale bar: 100 μm. (G) Quantification of Ki67- and caspase-3-positive cells and neutrophils as a percentage of total cells in the tumors. The percentage of positive cells in each tumor was calculated using ImageJ software. Data are presented as mean ± SEM. *P < 0.05, **P < 0.01, ***P < 0.001

The effect of CatX inhibition and simultaneous inhibition of cathepsins B and X on tumor growth and metastasis was investigated in the FVB/PyMT transgenic mouse model, which spontaneously develops numerous mammary tumors and lung metastases. In this model, numerous tumors spontaneously formed around the mammary glands of the mice, which prevented continuous measurement of the size of each tumor. Therefore, at the end of the experiment, the tumors were dissected and weighted to determine the tumor burden for each group. Compared to the control group, tumor weight was significantly reduced after treatment with **Z9** (67.18 mg/kg), nitroxoline (40 mg/kg), and co-treatment (Fig. 5A, B). However, co-treatment did not further decrease tumor weight compared to individual treatment with **Z9** or nitroxoline alone (Fig. 5A, B). Furthermore, compared to untreated mice, **Z9**-treated, nitroxoline-treated, and co-treated mice exhibited a significant reduction in the number and size of lung metastases (Fig. 5C). However, co-treatment also did not further reduce the number or size of metastases compared to treatment with a single inhibitor.

The body weight of the mice did not decrease throughout the course of the experiment. **Z9**, nitroxoline, or their combination did not induce systemic toxicity, as assessed by monitoring the body weight of the mice (data not shown).

Next, the effect of CatX and CatB inhibition was further investigated in the orthotopic breast cancer mouse model, in which primary MMTV-PyMT tumor cells were injected into the left inguinal mammary gland of FVB mice to form tumors. The doses of inhibitors received by these model mice were twofold lower than those in the transgenic mice. Tumor growth was significantly reduced by **Z9** (33.59 mg/kg), nitroxoline (20 mg/kg), and nitroxoline (20 mg/kg) and **Z9** (33.59 mg/kg) (Fig. 5D, E). Also, here, no additional reduction in tumor growth was observed after co-treatment compared to a single inhibitor (Fig. 5D).

### CatX inhibition affects tumor cell proliferation and death and neutrophil infiltration

Next, we analyzed the effects of cathepsin inhibitors on tumor progression, such as cell proliferation and death and neutrophil infiltration (Fig. 5F). Cell proliferation in tumors from the orthotopic breast cancer mouse model was determined by immunohistochemical detection of the proliferation factor Ki67. The data show that the percentage of Ki67-positive cells was significantly lower in the tumors of mice that received **Z9** or nitroxoline compared to those in the control group (Fig. 5G). Although co-treatment decreased the percentage of Ki67-positive cells, the difference was not significant compared to the control group (Fig. 5G).

The effect of treatment on cell death was assessed by quantifying the expression of caspase 3, a protein associated with the execution of apoptosis. Although the fraction of caspase-3-positive cells was low, their amount was significantly increased by more than twofold in tumors from **Z9**-treated, nitroxoline-treated, or co-treated mice (Fig. 5G). Furthermore, the presence of neutrophils in the tumors was examined. Similar to caspase 3, the percentage of neutrophils in the tumors was very low, accounting for approximately 1% of all cells in the untreated tumors. However, this percentage significantly increased in the tumors of **Z9**-treated (by ~ 2.4-fold), nitroxoline-treated (by ~ 2.4-fold), or co-treated (by ~ 1.7-fold) mice (Fig. 5G).

## Discussion

In this study, we showed that CatX inhibition is a viable strategy to improve antitumor therapy. We demonstrated that the CatX inhibitor **Z9** impairs processes of tumor progression associated with increased CatX activity in in vitro cell-based assays and tumor growth and progression in vivo in two independent tumor mouse models. Furthermore, we showed that CatX inhibition in combination with CatB inhibition further decreases tumor growth and progression in multiple independent in vitro models.

Increased CatX activity in cancer may contribute to tumor progression through proteolytic cleavage of substrates or by binding to various targets primarily involved in tumor migration, invasion, and adhesion. CatX enhances tumor cell migration and invasion through interaction with integrin receptors or cleavage of the C-terminal part of the tumor suppressor protein profilin-1. Interestingly, CatX can enhance tumor cell migration by interacting with integrin receptors and switching the migration mode from mesenchymal- to ameboid-like, which is independent of ECM degradation [6]. Inhibition of these processes by **Z9** was investigated in the current study using several independent assays and different tumor cell lines. For this purpose, **Z9** was used at a low micromolar concentration (5 μM) to avoid potential cytotoxic effects on the cells used.

**Z9** inhibited the migration of MCF-10A neoT and MMTV-PyMT cells in the scratch assay and the migration of MCF-10A neoT cells in the real-time assay using the xCELLigence system. MCF-10A neoT cells were used as model cell line as their transfection with c-Ha-ras oncogene results in highly malignant cell phenotype. Also, they are well characterized regarding their proteolytic profile and express high levels of both cathepsins [24, 36, 37]. It is evident that **Z9** affects different aspects of tumor cell migration, as interactions between cells are important in the scratch assay [38], whereas cells are not bound to the surface and migrate individually in all dimensions in the xCELLigence migration system. Both these aspects are important for the migration of tumor cells into surrounding tissues and to distant locations in vivo.

Next, two independent cell-based assays were applied to monitor tumor cell invasion. First, **Z9** reduced tumor cell invasion through a Matrigel layer, as monitored using the xCELLigence system. Second, **Z9** significantly reduced the invasive potential of MMTV-PyMT spheroids, observed as their reduced growth in the Matrigel. The first model monitors single-cell invasion using a 2D model of the ECM, while spheroids represent a 3D invasion model in vitro that more accurately represents the tumor microenvironment and its complexity, taking into account cell–cell interactions and cell organization [39]. Another mechanism by which CatX contributes to tumor progression is by interacting with integrin receptors, which modulates tumor cell adhesion [6, 9]. CatX modulates integrin receptors by subsequent proteolytic cleavage of up to four amino acids at C-terminal part of β2 chain of integrin receptors. This cleavage regulates the conformation of β2 integrin extracellular domain and consequently the binding to ECM components and other ligands, leading to increase in tumor cell adhesion [6, 21]. Consistent with this, we demonstrated that **Z9**-induced CatX inhibition significantly reduces tumor cell adhesion.

Next, the antitumor properties of **Z9** were investigated in two independent tumor mouse models. **Z9** exhibited antitumor activity in the FVB/PyMT transgenic mouse model that spontaneously forms mammary tumors and lung metastases [31]. Administration of **Z9** (67.18 mg/kg) reduced tumor weight and the number and size of lung metastases, suggesting that **Z9** also exerts anti-metastatic activity. Reduced tumor growth was also demonstrated in the orthotopic mouse breast cancer model established by injecting primary MMTV-PyMT cells into the left inguinal mammary gland of FVB recipient mice [32]. Although in this model, **Z9** (33.59 mg/kg) was used at half the dose of that used in the transgenic model (67.18 mg/kg) assess more precisely the effect of CatX, it still significantly reduced tumor growth. The resected tumors from the orthotopic breast cancer mouse model were examined for cell proliferation and death and neutrophil infiltration by immunohistochemically detecting proliferation factor Ki67, caspase 3, and neutrophil infiltration, respectively. The results showed a significant decrease in the proliferation rate of **Z9**-treated tumors compared to the other groups, which is in line with the decreased tumor growth in this cohort of mice. Taken together, these results demonstrate potent antitumor activity of **Z9** and further validate CatX inhibition as a viable strategy to impair tumor growth and progression.

An important feature of CatX during cancer progression is its ability to compensate for the loss of CatB activity. CatB appears to be a major tumor promoter among the cysteine cathepsins and is, besides CatX, the only cysteine cathepsin that acts as a carboxypeptidase in lysosomes. CatB also acts as an endopeptidase at higher pH due to a conformational change caused by a structural element termed the occluding loop, which regulates the access of substrates to the active site cleft [40, 41]. CatB is overexpressed in all types of tumors, and inhibition of its expression has been frequently reported to reduce tumor growth and progression in vitro and in vivo [36, 40, 42, 43]. The compensatory effect of CatX over CatB was first shown in CatB/CatX knockout mice [21, 22]. Also, in the current study, we demonstrated increased CatX activity and protein levels after CatB inhibition, confirming that CatX activity is indeed responsible for resistance developed after CatB-targeted antitumor therapy.

For CatB inhibition, we used nitroxoline, a well-established antimicrobial agent, which we have identified as a potent, selective, reversible inhibitor of CatB endopeptidase activity with strong antitumor activity in vitro and in vivo [36, 44]. Antitumor activity of nitroxoline was independently demonstrated by multiple other studies, where especially its antiangiogenic properties by inhibiting type 2 methionine aminopeptidase and sirtuin-1 [45] were highlighted, together with its ability to induce apoptosis and cell-cycle arrest (reviewed in [46]). Similarly to the action of the single inhibitors nitroxoline and **Z9**, the combined use of nitroxoline and **Z9** significantly affected tumor cell migration, invasion, and adhesion in all the assays tested. Moreover, a synergistic effect in the inhibition of tumor cell migration was detected after co-treatment compared to individual inhibitors. This effect was particularly pronounced when tumor cell migration was monitored by scratch assay. Similarly, co-treatment also exerted a stronger effect on the growth of tumor spheroids compared to individual inhibitors. On the other hand, a moderate additive effect of both compounds over each individual compound was observed in the invasion and adhesion assays when monitored continuously.

Both inhibitors were also tested for potential synergistic effects in vivo in MMTV-PyMT transgenic and orthotopic breast cancer tumor mice [21, 22, 32]. In both mouse models, the co-treatment with nitroxoline and **Z9** had a potent effect on tumor weight and the number and size of lung metastases. In the orthotopic breast cancer mouse model, co-treatment also decreased cell proliferation (the number of Ki67-positive cells), increased caspase 3 expression in tumors, and increased neutrophil infiltration into the tumors. However, in both tumor mouse models, no additive effect of co-treatment was observed regarding tumor growth, metastasis, cell proliferation, apoptosis, or neutrophil infiltration, compared to treatment with one inhibitor.

Our results suggest that the synergistic effect of simultaneous inhibition of cathepsins X and B is more evident in processes less dependent on EMC degradation, since CatX does not contribute much to this process due to its solely carboxypeptidase activity. **Z9**-induced CatX inhibition had no effect on the degradation of DQ-collagen IV, either extracellularly or intracellularly, whereas nitroxoline and the combination of both inhibitors significantly diminished ECM degradation. The fact that nitroxoline alone and in combination with **Z9** inhibited ECM degradation confirms the involvement of CatB, but not CatX, in this process. The role of CatX in cell–cell and cell–ECM interactions as well as cytoskeleton remodeling, especially by modulation of integrin receptors, may have a greater impact on tumor cell migration than on invasion. Since CatX promotes the ameboid mode of T lymphocyte migration [47], its role in a switch between mesenchymal and amoeboid modes of tumor cell migration is to be expected. On the other hand, CatB, that acts as endopeptidase, degrades ECM and promotes mesenchymal type cell migration, while despite extensive studies its impact on integrin receptors is less known and it does not seem to be crucial for tumor promotion [40–43]. Considering these differences in mechanisms between two cathepsins, the focus of the study was given to CatB dependent targets and processes. Also, the compensatory effect between cathepsins B and X in tumor angiogenesis is less likely to be seen due to the action of nitroxoline on multiple targets.

The experimental setting might also limit the synergistic effect of both inhibitors in in vivo tumor mouse models. Some CatX-specific functions, such as modulating the immune system, were apparently not evident during the observation period. The impact on possible immune processes was examined on neutrophils infiltration. However, results do not confirm the contribution of both inhibitors on neutrophil function. In future studies, the neutrophil status might be examined in detail, including the immunodetection of different types of neutrophils (N1, N2, TAN), local cytokine-chemokine status, and the neutrophil-to-lymphocyte ratio. Furthermore, the detection of protumorigenic macrophages such as CD 204 + should also be included in the evaluation of tumor aggressivity. Furthermore, co-treatment started at the same time; however, in further experiments, the CatX inhibitor should be applied with a time delay as its activity and expression increases as a consequence of CatB inhibition. Furthermore, optimization of the treatment regimen and dosage is also required to achieve the optimal effects of both inhibitors in vivo.

In summary, we have shown that **Z9**, a triazole-based selective reversible inhibitor of CatX, possesses potent antitumor activity in vitro (in cell-based functional assays mimicking the main processes of tumor progression) and in vivo (in two independent tumor mouse models). Moreover, we confirmed that CatB inhibition is followed by an increase in CatX activity and protein levels that can compensate for the loss of CatB function. The simultaneous inhibition of both cathepsins B and X showed a synergistic effect in in vitro assays of tumor cell migration and spheroid growth, whereas such co-treatment requires further optimization in in vivo experiments. Importantly, novel CatX inhibitors enable the investigation of the complex proteolytic interplay of cathepsins in cancer, as well as in neurodegenerative and immune diseases associated with dysregulated CatX function.

Results obtained during this study open new possibilities for future work. For example, the mechanism and the impact of CatX inhibition on downstream molecular targets of CatX would be worth to investigate. Next, the effect of CatX inhibition as well as concurrent inhibition of both cathepsins B and X on tumor angiogenesis and antitumor immune response would also be interesting to evaluate. Moreover, the optimization of the best treatment regimen and dosage to achieve the best effect of both inhibitors, especially in vivo, is required as well. All these aspects could further expand the knowledge on the proteolytic system and its role in cancer.

## Supplementary Information

Below is the link to the electronic supplementary material.

Supplementary file1 (DOCX 180 KB)

## Data Availability

All data generated during this study are included in this published article.

## Abbreviations

CatB: Cathepsin B
CatX: Cathepsin X
CI: Cell index
DMEM: Dulbecco’s modified Eagle’s medium
ECM: Extracellular matrix
FBS: Fetal bovine serum
MMTV-PyMT: Mouse mammary virus-polyoma middle T antigen
PET: Polyethylene terephthalate
PyMT: Polyoma middle T
