# Supplementary material for: Evaluation of novel cathepsin-X inhibitors in vitro and in vivo and their ability to improve cathepsin-B-directed antitumor therapy
Source: Cell Mol Life Sci. 2022 Jan 6;79(1):34. doi: 10.1007/s00018-021-04117-w (PMC8738504; doi:10.1007/s00018-021-04117-w)
Supplement: Supplementary file 1 — Supplementary file1 (DOCX 180 KB) [file 18_2021_4117_MOESM1_ESM.docx]

# Supplementary information

# Evaluation of novel cathepsin X inhibitors *in vitro* and *in vivo* and their ability to improve cathepsin B-directed antitumor therapy

Ana Mitrović^1,*^, Janja Završnik^2^, Georgy Mikhaylov^2^, Damijan Knez^3^, Urša Pečar Fonović^3^, Petra Matjan Štefin^2^, Miha Butinar^2^, Stanislav Gobec^3^, Boris Turk^2,4^, Janko Kos^1,3^

^1^Department of Biotechnology, Jožef Stefan Institute, Ljubljana, Slovenia

^2^Department of Biochemistry and Molecular Biology, Jožef Stefan Institute, Ljubljana, Slovenia

^3^Faculty of Pharmacy, University of Ljubljana, Ljubljana, Slovenia

^4^Faculty of Chemistry and Chemical Technology, University of Ljubljana, Ljubljana, Slovenia

E-mail addresses: [ana.mitrovic@ijs.si](mailto:ana.mitrovic@ijs.si) (A. Mitrović)

^*^Corresponding author:

Ana Mitrović, Department of Biotechnology, Jožef Stefan Institute, Jamova 39, 1000 Ljubljana, Slovenia, [ana.mitrovic@ijs.si](mailto:ana.mitrovic@ijs.si)

## Supplementary Figures


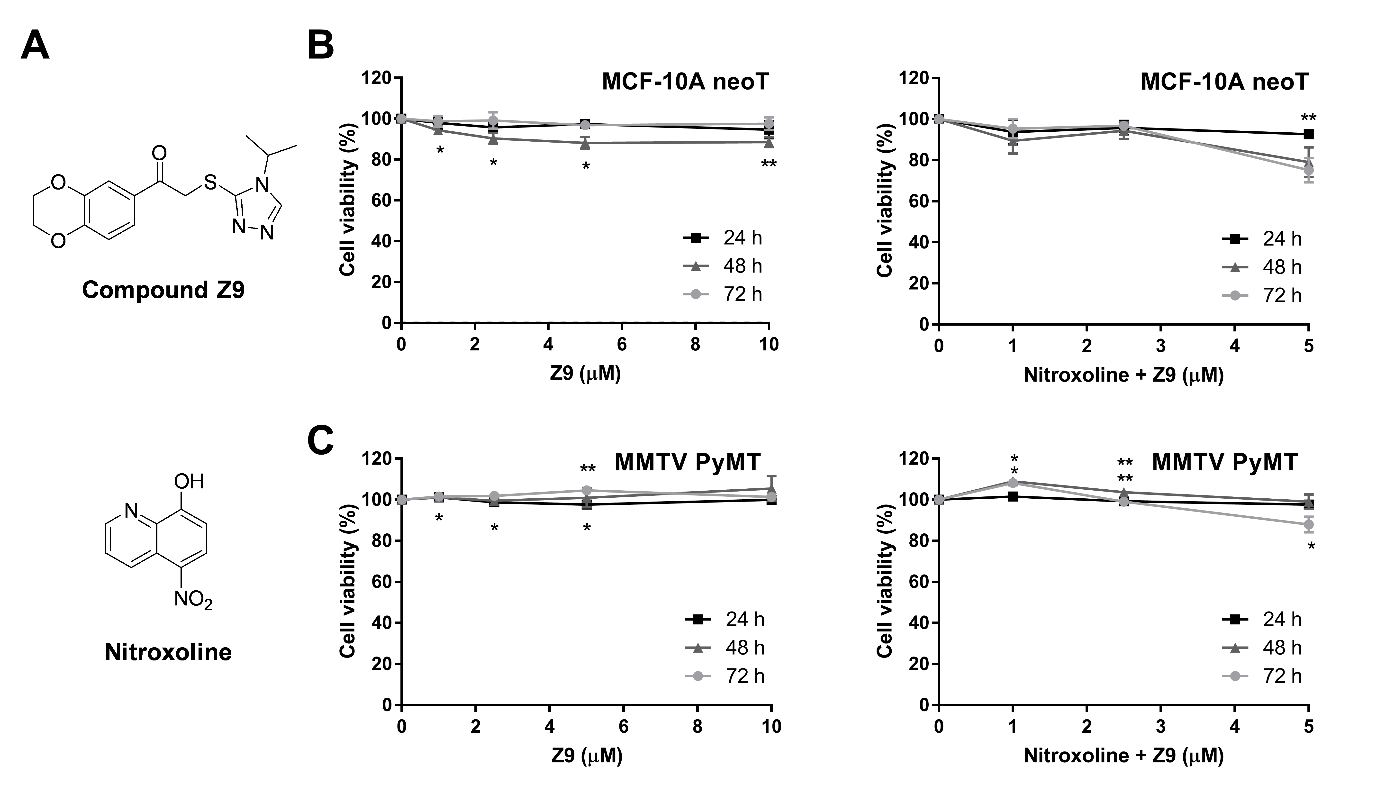


**Supplementary Figure 1.** The effect of **Z9** and co-treatment with both **Z9** and nitroxoline on cell viability. (A) Structures of the CatX inhibitor **Z9** and the CatB inhibitor nitroxoline. (B) MCF-10A neoT cells (3 × 10^4^ for 24 h, 1 × 10^4^ for 48 h, or 5 × 10^3^ for 72 h) and (C) MMTV-PyMT cells (3 × 10^4^ for 24 h, 1 × 10^4^ for 48 h, or 5 × 10^3^ for 72 h) were treated with increasing concentrations of **Z9** or nitroxoline and **Z9** together for 24, 48, or 72 h. Cell viability was determined by the MTS assay. Data are presented as percentage of viable cells (mean ± SEM) from at least two independent experiments, each performed in quadruplicate. **P* < 0.05, ***P* < 0.01.

## Supplementary Methods

**Whole cell lysate preparation**

Whole cell lysates were prepared in 50 mM Na acetate, pH 5.5, 1 mM EDTA, 100 mM NaCl, and 0.25% Triton X-100 lysis buffer. For western blot analysis, the lysis buffer was supplemented with cOmplete™ Protease Inhibitor Cocktail (Roche, Basel, Switzerland) and PhosSTOP™ (Rosche) as phosphatase inhibitor cocktail. Cell lysates were centrifuged at 16800×g at 4 °C for 20 min, and the supernatant was collected. Protein concentration was determined using the DC Protein Assay Kit (Bio-Rad, Hercules, CA, USA).

**Determination of cathepsin X activity**

Cathepsin X (CatX) activity was assessed using the specific fluorogenic substrate Abz-Phe-Glu-Lys (Dnp)-OH (synthesized by Nace Zidar, Faculty of Pharmacy, University of Ljubljana, Ljubljana, Slovenia according to [1]). The substrate (10 μL, 10 μM) was added to the wells of a black microplate, and the reaction was started with the addition of 90 μL of lysate in assay buffer (100 mM acetate buffer (pH 5.5) containing 0.1% polyethylene glycol (PEG) 8000, 5 mM cysteine, and 1.5 mM EDTA). The samples were activated in the assay buffer for 5 min at 37 °C prior to assay. The formation of the fluorescent degradation product was monitored continuously during the reaction at 420 nm ± 10 nm with excitation at 320 nm ± 20 at 37 °C on a Tecan Infinite M1000. The assay was performed in duplicates and repeated four times.

**Western blot analysis**

Equal amounts of protein (20 μg of total proteins per lane) were separated by SDS-PAGE on 12% gels and transferred to a nitrocellulose membrane using the Trans-Blot Turbo system (Bio-Rad). To block nonspecific binding, membranes were first blocked with 1.5% (w/v) nonfat dry milk powder and 1% bovine serum albumin in TBST (0.1% Tween 20 in Tris-buffered saline, pH 7.4) for 1 h at room temperature (RT). The membranes were then incubated with primary antibodies overnight at 4 °C or for 1 h at RT. Afterwards, the membranes were washed with TBST and incubated with conjugated secondary antibodies for 1.5 h at RT. Bands were visualized using Clarity™ Western ECL Substrate (Bio-Rad) on a ChemiDoc MP Imaging System (Bio-Rad). The following primary and secondary antibodies were used: goat anti-CatX (1:500, AF934, R&D), rabbit anti-β-actin (1:3000, A2066, Sigma), mouse anti-goat IgG horseradish peroxidase (HRP) (1:3000, sc-2354, Santa Cruz Biotechnology), and goat anti-rabbit DyLight 650 (1:5000, SA-10034, Invitrogen). Four independent replications of the assay were performed.

**Cell viability assay**

The effect of **Z9** and co-treatment with nitroxoline and **Z9** on the viability of MCF-10A neoT and MMTV PyMT cells was evaluated using the MTS [3-(4,5-dimethylthiazol-2-yl)-5-(3-carboxymetoxyphenyl)-2-(4-sulfophenyl)-2*H*-tetrazolium] colorimetric assay (CellTiter 96 Aqueous One Solution Cell Proliferation Assay, Promega, Madison, WI, USA). The cells were seeded at 3 × 10^4^ (for 24 h), 1 × 10^4^ (for 48 h), or 5 × 10^3^ (for 72 h) cells/well into a 96-well microplate and incubated overnight for attachment. The cells were then treated with the inhibitor (at 1, 2.5, or 5 µM) or DMSO (0.1%) in 100 μL for 24, 48, or 72 h. After incubation, 10 µL of the reagent MTS was added to the wells, and the absorbance of formazan was measured at 492 nm on a Tecan Infinite M1000 (Mannedorf, Switzerland). Cell viability (%) was expressed as the ratio between absorbance in the presence of the compounds and in the presence of DMSO. All assays were performed in quadruplicate and repeated at least twice.

**DQ-collagen IV degradation assay**

DQ-collagen IV was used to observe the effect of cathepsin B and X inhibition on EMC degradation. Intracellular degradation of DQ-collagen type IV was monitored by flow cytometry. MCF-10A neoT cells were seeded at 6 × 10^4^ cells/well into a 24-well plate and allowed to adhere overnight at 37 °C. The cells were then treated with **Z9** (50 µM), nitroxoline (50 µM), both compounds together, or DMSO (0.5%) in 500 μL serum-free medium (SFM) for 2 h at 37 °C. After 2 h of incubation, DQ-collagen IV (5 μg/mL; Thermo Fischer, Rockford, IL, USA) was added, and the cells were incubated for an additional 2 h at 37 °C. The cells were detached and washed with phosphate-buffered saline (PBS). Propidium iodide (10 μg/mL; BD Bioscience) was added to exclude dead cells, and thus green fluorescence resulting from the degradation of DQ-collagen IV was monitored only for viable cells. The measurement was performed using the FACSCalibur system (BD Biosciences). The assay was performed in duplicates and repeated four times.

Extracellular degradation of DQ-collagen IV was monitored with spectrofluorimetry. MCF-10A neoT cells were seeded at 3 × 10^4^ cells/well into a 96-well microplate and allowed to adhere overnight at 37 °C. The next day, the medium was replaced with 100 μL of fresh medium containing **Z9** (5 µM), nitroxoline (5 µM), nitroxoline and **Z9** together (5 µM each), or DMSO (0.1%). The cells were incubated for 24 h, and then the medium was removed. Next, 100 μL of PBS containing DQ-collagen IV (10 μg/mL) and either the inhibitor **Z9** (5 µM), nitroxoline (5 µM), nitroxoline and **Z9** together (5 µM each), or DMSO (0.1%) was added and incubated at 37 °C for 6 h. After incubation, the reaction mixture (80 μL) was transferred to empty wells of a black 96-well microplate, where fluorescence intensity was continuously monitored for 2 h at 515 nm ± 5 nm with excitation at 495 nm ± 5 nm on a Tecan Infinite M1000 microplate reader. Inhibition of extracellular DQ-collagen IV degradation was expressed as the ratio between the average 2 h fluorescence obtained in the presence of the compounds and DMSO. The assay was performed in six parallels and repeated two times.

**Real-time migration assay**

Tumor cell migration was also monitored in real-time with an xCELLigence Real-Time Cell Analyzer (RTCA; Agilent, Santa Clara, CA, USA). The system continuously tracks tumor cell migration through the microporous membrane separating the top and bottom chambers by measuring impedance, expressed as CI, across the microelectrodes integrated into the membrane. Cells were serum-starved for 24 h before the assay. The experiment was performed on the Cell Invasion and Migration (CIM)-plate 16 (Agilent). First, the lower and upper sides of the polyethylene terepthalate (PET) membranes on the CIM-plate 16 were coated with 10 µg/mL fibronectin from bovine plasma for 30 min at RT and 2 h at 37 °C, respectively. Excess fibronectin was removed, and the wells were washed with PBS. Then, 160 µL of the complete medium containing the compounds **Z9** (5 µM), nitroxoline (2.5 µM), nitroxoline (2.5 µM) and **Z9** (5 µM) together, or DMSO (0.1%) was added to the lower chamber. Both parts of the plate were assembled together, 60 µL of the compounds (2.5 or 5 µM) or DMSO (0.1%) in SFM was added to the upper chamber, and the CIM-plate 16 was equilibrated for 1 h at 37 °C. Next, MCF-10A neoT cells (in 100 µL of SFM) were seeded at 2 × 10^4^ cells/well into the upper chambers of the CIM-plate 16 and inserted into the xCELLigence system for data collection. The cell index (CI), which represents the relative change in electrical impedance, was recorded in real time every 15 min for 72 h. RTCA Software (Roche) was used to analyze the data. Relative migration was expressed as a percentage relative to DMSO-treated control cells. The assay was performed in triplicates and repeated at least three times.

**Real-time invasion assay**

The effect of cathepsin B and X inhibition on MCF-10A neoT tumor cell invasion was monitored with the xCELLigence RTCA. The cells were serum-starved for 24 h prior to the experiment, which was performed on a CIM-plate 16. First, the bottom side of the microporous PET membrane was coated with 0.3 μg of fibronectin from bovine plasma (Sigma) for 30 min at RT. Then, the upper chambers of the CIM-plate 16 wells were coated with Matrigel (20 μL; 1 mg/mL; BD Biosciences, Franklin Lakes, NJ, USA) in SFM and allowed to gel at 37 °C for 30 min. Next, 160 µL of the compounds **Z9**, nitroxoline, nitroxoline and **Z9** together (all 5 µM), or DMSO (0.1%) in complete medium were added to the lower chambers. Then the top and bottom chambers of the CIM-plate 16 were assembled together. Next, 60 µL of the compounds (5 µM) or DMSO (0.1%) in SFM was added to the upper chambers of the CIM-plate 16, which was then equilibrated for 1 h at 37 °C. Afterwards, MCF-10A neoT cells (in 80 µL of SFM) were seeded at 3 × 10^4^ cells/well into the upper chambers of the CIM-plate 16 and inserted into the xCELLigence system for data collection. The CI was recorded in real time every 15 min for 72 h. RTCA software was used to analyze the data. Relative invasion was expressed as a percentage relative to DMSO-treated control cells. The assay was performed in triplicates and repeated four times.

**Real-time adhesion assay**

The effect of cathepsin B and X inhibition on the adhesion of MCF-10A neoT cells to the microelectrodes installed at the bottom of the E-plate View 16 PET plates (Agilent) was monitored using xCELLigence RTCA. The wells were first coated with 10 µg/mL fibronectin for 1 h at 37 °C and then washed with PBS. Next, 100 µL of complete medium containing the compounds **Z9** (5 µM), nitroxoline (2.5 µM), nitroxoline (2.5 µM) and **Z9** (5 µM) together or DMSO (0.1%) was added. After equilibrating the plate for 45 min at 37 °C, MCF-10A neoT cells were seeded at 5 × 10^3^ cells/well (in 100 µL of complete medium), and the plate was placed into the xCELLigence system for data acquisition. CI values were monitored in real time every 15 min for 72 h, and data were analyzed using the RTCA software. Relative cell adhesion was expressed as a percentage relative to DMSO-treated control cells. The assay was performed in triplicates and repeated at least two times.

**Three-dimensional invasion assay**

The spheroids were prepared according to the hanging drop method [2]. To prepare the spheroids, 20 µL drops of MMTV-PyMT cell suspension (250 cells/drop) were placed onto the lids of 100 mm tissue culture dishes, which were then inverted over 10 mL of water. After 5 days, the formed aggregates were transferred to the wells of a 96-well black μCLEAR transparent flat bottom plate (Greiner Bio-One, Kremsmuenster, Austria) coated with 30 µL Matrigel (5 mg/mL) in SFM. Subsequently, the spheroids were covered with another 30 µL of Matrigel. After 20 min of incubation at 37 °C, 200 µL of complete medium was added. Compounds (5 µM) or DMSO (0.1%) were added to the Matrigel and medium. Spheroid growth was monitored daily for up to 3 days by measuring the spheroid dimensions under the light microscope, using an ocular micrometer. The spheroid volume was calculated according to the following equation: *V = (π × (spheroid length) × (spheroid width)^2^)/6*. The experiment was repeated three times and for each experiment dimensions of at least three spheroids were monitored for each individual treatment condition. Representative images of tumor spheroids were acquired using the AxioObserver Z1 fluorescence inverted microscope (Carl Zeiss, Oberkochen, Germany) and AxioVision 4.8.2 software (Carl Zeiss, Oberkochen, Germany).

**Immunohistochemistry**

The tumors were harvested and fixed overnight in 10% neutral buffered formalin and then processed for paraffin embedding. Formalin-fixed, paraffin-embedded tumor specimens were sectioned (5 µm) and mounted onto glass slides. Slides were deparaffinized and rehydrated through graded alcohol to distilled water, followed by antigen retrieval with 10 mM sodium citrate, 0.05% Tween 20, pH 6.0. Endogenous peroxidase was quenched with 0.3% hydrogen peroxide. Sections were stained with antibodies against Ki67 (M724901, rabbit monoclonal, dilution 1:50; Dako, Carpinteria, CA, USA), Ly-6G/Ly-6C (a neutrophil marker; RB6-8C5, rat monoclonal, dilution 1:50; Thermo Fisher Scientific, MA, USA), and active caspase 3 (ab2302, rabbit polyclonal, dilution 1:200; Abcam, Cambridge, UK). Detection was performed using the Vectastain Elite ABC kit (Vector Laboratories, Inc., Burlingame, CA, USA), and staining was visualized with 3,3′-diaminobenzidine (DAB; Sigma-Aldrich). Slides were counterstained with hematoxylin, dehydrated, cleared, and coverslipped. Stained sections were imaged using an IX81 brightfield microscope (Olympus, Tokyo, Japan) and Imaging Software for Life Science Microscopy Cell^F (Olympus). For data analysis, 20 fields per tumor were randomly selected with the 40× objective and quantified using ImageJ software.

For volumetric measurements of lung metastases, excised lungs were cut transversally to the trachea into systematic 5-mm-thick slabs (n = 5–7), which were embedded with paraffin into a single block. Next, 5-μm-thick paraffin sections from each block were stained with hematoxylin and eosin. Stereological determination of the metastatic volumes was performed using an IX81 bright-field microscope and Imaging Software for Life Science Microscopy Cell^F.

**References**

1. Puzer L, Cotrin SS, Cezari MHS, et al (2005) Recombinant human cathepsin X is a carboxymonopeptidase only: A comparison with cathepsins B and L. Biol Chem 386:1191–1195. https://doi.org/10.1515/BC.2005.136

2. Kelm JM, Timmins NE, Brown CJ, et al (2003) Method for generation of homogeneous multicellular tumor spheroids applicable to a wide variety of cell types. Biotechnol Bioeng 83:173–180. https://doi.org/10.1002/bit.10655
